# Clinical relevance of partial HPV16/18 genotyping in stratifying HPV‐positive women attending routine cervical cancer screening: a population‐based cohort study

**DOI:** 10.1111/1471-0528.16631

**Published:** 2021-01-12

**Authors:** S Gori, J Battagello, D Gustinucci, C Campari, M Zorzi, H Frayle, B Passamonti, G Sartori, S Bulletti, C Fodero, E Cesarini, R Faggiano, A Del Mistro

**Affiliations:** ^1^ Immunology and Diagnostic Molecular Oncology Unit Veneto Institute of Oncology IOV‐IRCCS Padua Italy; ^2^ Veneto Tumour Registry Azienda Zero Padua Italy; ^3^ Laboratorio Unico di Screening USL Umbria 1 Perugia Italy; ^4^ Cancer Screening Unit Azienda Unità Sanitaria Locale ‐ IRCCS di Reggio Emilia Reggio Emilia Italy; ^5^ Laboratorio citologia cervico‐vaginale Azienda Unità Sanitaria Locale ‐ IRCCS di Reggio Emilia Reggio Emilia Italy

**Keywords:** Cervical cancer screening, CIN3, genotyping, HPV, triage

## Abstract

**Objective:**

To evaluate partial HPV16/18 genotyping as a possible biomarker to select women attending HPV‐based cervical cancer screening at higher risk to be referred to colposcopy.

**Design:**

Population‐based cohort study.

**Setting:**

Organised cervical cancer screening programmes (Italy).

**Population:**

Women with high‐risk HPV infection (period: 2015–2019).

**Methods:**

We analysed the association between partial HPV16/18 genotyping, cytology triage and histologically confirmed diagnosis of high‐grade cervical intraepithelial neoplasia (CIN3^+^) lesions.

**Main outcome measures:**

Detection rate (DR) and positive predictive value (PPV) for histologically confirmed CIN3^+^ (any episode in the 2 years after baseline); sensitivity for CIN3^+^ and number of colposcopies needed for lesion detection.

**Results:**

The study included 145 437 women screened with HPV testing by the clinically validated COBAS 4800 HPV assay (Roche). Overall, 9601 (6.6%) women were HPV^+^ at baseline; HPV16 and HPV18 were present in 1865 and 594 samples, respectively. The cumulative (baseline plus 1‐year repeat) cytology positivity was 42.8% and high‐grade cytology was significantly higher (*P* < 0.0001) among women with HPV16 infection at baseline (15.2%). The cumulative CIN3^+^ DR for women with HPV16, HPV18 and other HPV‐type infections was 9.8%, 3.4% and 1.8%, respectively.

**Conclusions:**

Partial HPV16 genotyping may play a role in triage, whereas HPV18 seems to behave much more similarly to the other HPV types and does not provide additional stratification. HPV16 genotyping combined with high‐grade cytology can be envisaged as a triage biomarker in cervical screening to maximise CIN3^+^ detection while minimising colposcopy at baseline or 1‐year repeat.

**Tweetable abstract:**

HPV16 genotyping combined with high‐grade cytology can be used as triage biomarker for CIN3^+^ in HPV‐positive women.

## Tweetable abstract

HPV16 genotyping combined with high‐grade cytology can be used as a triage biomarker for CIN3^+^ in HPV‐positive women.

## Introduction

Cervical cancer is caused by persistent infection with high‐risk human papillomaviruses (HPV),^1^, ^2^ and genotypes HPV16 and HPV18 cause approximately 70% of the global cervical cancer cases.^3^

Screening tests are used to identify women at risk of developing cancer and aim to reduce the risk of disease and associated mortality by detecting and treating precursor lesions before they progress to cervical cancer.^4^

European guidelines recommend primary HPV testing for organised, population‐based screening.^5^

In Italy, the implementation of organised cervical screening programmes has been recommended since 1996,^6^ and screening has been included in the Ministry of Health's list of ‘*Essential Health Interventions*’ since 2001.^7^ Organised screening programmes are implemented at a regional level and are based on call‐and‐recall invitation of all women aged 25–64 years, and systematic monitoring of the indicators set by the Ministry of Health is performed annually.

In 2013, following the results of pilot projects including 25‐ to 64‐year‐old or 35‐ to 64‐year‐old women, the Italian Ministry of Health included HPV test every 5 years as an option for screening programmes for women aged ≥30 years.^8^ The National Prevention Plan 2014–2018 set as an objective the full implementation of HPV‐based screening by 2018;^9^ timing of this implementation differs by region.

HPV infections have a transient nature, with only a small proportion of HPV‐positive women developing high‐grade cervical intraepithelial neoplasia (CIN).^10^, ^11^

Therefore, all HPV‐positive results need additional triage in order to identify women with a high risk of cervical cancer and precancer (CIN3^+^). Cytology is the most frequently used triage strategy, but the management of HPV‐positive women with negative cytology is particularly challenging, as the risk of disease is too high to return these women to regular screening but too low for colposcopy referral,^12^, ^13^, ^14^, ^15^ and needs additional testing.

Multiple studies have evaluated triage strategies for HPV‐positive women in large screening cohorts.^16^, ^17^, ^18^, ^19^, ^20^, ^21^, ^22^ Strategies with immediate cytology, HPV16/18‐genotyping, repeat HPV testing and/or repeat cytology have been identified and adopted in current HPV‐screening guidelines.

We analysed the association between partial 16/18 genotyping of HPV‐positive tests with triage cytology and histologically confirmed diagnosis of high‐grade lesions in women attending three population‐based cervical screening programmes in order to investigate the usefulness of genotyping as triage test.

## Methods

### Study population

Women attending organised population‐based cervical cancer screening by HPV testing in three different regions of North (Veneto and Emilia‐Romagna) and Central (Umbria) Italy were included in the study.

Veneto and Emilia‐Romagna participated with one of the three regional centralised HPV laboratories, whereas Umbria with the only one regional laboratory. Organised cervical cancer screening has been in place in all three regions for more than 20 years; HPV testing has replaced cytology as the primary test since 2015.

**Figure 1:**
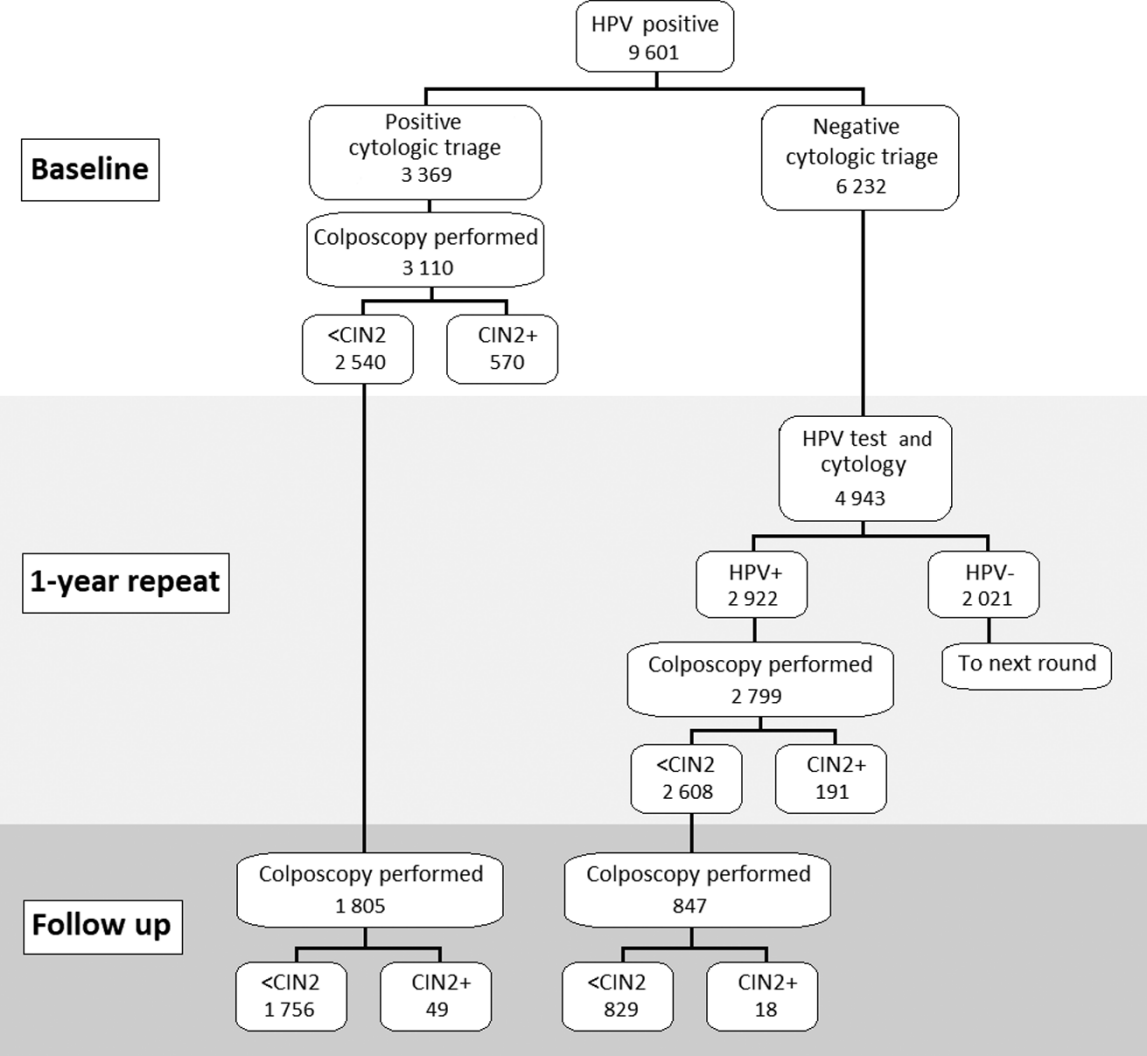
Flowchart of the study.

The Italian HPV‐screening protocol was applied to women older than 30 years and included HPV testing with cytology triage^23^ for HPV positives. Women with positive HPV (HPV^+^) and positive cytology (diagnosis of atypical squamous cells of undetermined significance (ASC‐US) or worse – cyto^+^) underwent immediate colposcopy; women with HPV^+^ and negative cytology (cyto^−^) were referred to HPV re‐testing at 1 year. In this paper, we refer to HPV testing plus cytology triage and eventual colposcopy as ‘baseline’. After 1 year, women with persistent infection underwent colposcopy (irrespective of cytology result), whereas those with a negative HPV returned to regular screening (i.e. they were invited for HPV testing after 5 years (Figure 1).

For women sent to colposcopy, in the case of negative colposcopy (see criteria in the following paragraph) women were followed by repeat testing, according to the local protocols, with timing between visits being defined according to cytology grade.

A 2‐year period of post‐colposcopy follow up (FUP) was available and was considered for this study. Despite the availability of national indications for the FUP protocol after an abnormal Pap test and a negative colposcopy, there is no uniformity in their application,^24^ in particular regarding the timing between control visits. For this reason, any episode of FUP following the baseline was evaluated, and the first episode with a diagnosis of CIN3^+^ was recorded.

Data about HPV test, cytology, colposcopy and histological diagnosis at baseline and 1‐year repeat were extracted by the screening programmes databases. As regards post‐colposcopy FUP, only data for HPV positivity and histological diagnosis were available.

### HPV test, cytology and colposcopy

HPV testing was performed by the clinically validated COBAS 4800 HPV assay (Roche Molecular Systems, Pleasanton, CA, USA), based on real‐time polymerase chain reaction (PCR) technology.

The method detects 14 HPV types (the 12 designed as high‐risk by the IARC, plus types 68 and 66) and provides specific results for HPV16 and HPV18, and altogether for the other 12 types (hereafter referred to as non16/18HPV), and includes an internal quality control (beta‐globin) for each sample. In the case of multiple infections, women with at least HPV16 (including HPV18) were classified as HPV16, and women with HPV18 or co‐infected by HPV 18 and other non‐16 types were classified as HPV18. Typing was not used for triage.

Cytology triage was classified according to the 2014 Bethesda reporting system: negative for intraepithelial lesion or malignancy (NILM) and epithelial cell abnormalities categorised in low grade (LG) and high grade (HG). LG includes ASC‐US and low‐grade squamous intraepithelial lesion (LSIL). HG includes atypical squamous cells, not excluding high‐grade SIL (ASC‐H), high‐grade squamous intraepithelial lesion (H‐SIL), atypical glandular cells favor neoplastic (AGC), adenocarcinoma in situ (AIS), squamous cell carcinoma and adenocarcinoma.^25^

Colposcopies were performed according to the 2011 colposcopic terminology of the International Federation for Cervical Pathology and Colposcopy (IFCPC).^26^ Cervix and squamocolumnar junction visibility, type of transformation zone (1, 2, 3) and grading of colposcopic findings (grade 1, minor; grade 2, major) were recorded. Biopsy was performed in the case of suspected lesions or high‐grade cytology. Endocervical curettage was performed in the case of glandular atypia.

### Statistical analysis

The primary outcomes of the study were (i) the prevalence of HPV types at baseline, (ii) the detection rate (DR) and positive predictive value (PPV) for histologically confirmed CIN3^+^ (any episode in the 2 years after baseline) of HPV16, HPV18 and non16/18HPV, and (iii) the sensitivity for CIN3^+^ and number of colposcopies needed for lesion detection. Histologically confirmed CIN2^+^ was a secondary outcome of the study.

The parameters listed below were calculated for HPV16, HPV18 and non16/18HPV groups.

### At baseline

- HPV test positivity (HPV^+^ tests/HPV tests).
- Proportion of positive cytology among HPV^+^ (women with cyto^+^ at baseline/HPV^+^ women), overall and by cytology grade (low, high).
- Detection of CIN3^+^ and CIN2^+^ among HPV^+^ (women with histologically confirmed CIN3^+^ (CIN2^+^) at baseline/HPV^+^ women).
- Positive predictive value (PPV) for CIN3^+^ and CIN2^+^ of biopsy (women with histologically confirmed CIN3^+^ (CIN2^+^) at baseline/biopsies performed).

### At 1‐year repeat

- Persistence of HPV positivity at 1‐year (HPV^+^ tests/HPV tests at repetition).
- Proportion of positive cytology at 1‐year repeat (women with cyto^+^ at 1‐year/ HPV tests at repetition), overall and by cytology grade (low, high).
- Detection of CIN3^+^ and CIN2^+^ (women with histologically confirmed CIN3^+^ (CIN2^+^) at 1‐year repeat/HPV tests at repetition).
- Positive predictive value (PPV) for CIN3^+^ and CIN2^+^ of biopsy (women with histologically confirmed CIN3^+^ (CIN2^+^) at 1‐year repeat/biopsies performed at repetition).

### Cumulative

- Detection of CIN3^+^ and CIN2^+^ (women with histologically confirmed CIN3^+^ (CIN2^+^) at baseline + 1‐year repeat/HPV tests at baseline).
- Positive predictive value (PPV) for CIN3^+^ and CIN2^+^ of biopsy (women with histologically confirmed CIN3^+^ (CIN2^+^) at baseline + 1‐year repeat/biopsies performed at baseline + 1‐year repeat).

For women with a persistent HPV infection at 1‐year, we compared the HPV type detected at baseline with the HPV type at repetition.

### At follow up

- Results for HPV testing.
- CIN3^+^ detection rate.

All analyses were performed for all screened women and (limited to Umbria and Veneto regions) stratifying the indicators in four age classes (25–29, 30–44, 45–54 and 55–64 years).

A sensitivity analysis was performed estimating the main outcomes (i.e. the baseline and cumulative detection of CIN3^+^) excluding all women who had multiple HPV types detected.

Finally, according to the study results, we identified different triage strategies of HPV positives at the baseline and for women with a persistent HPV at the 1‐year repeat. For each strategy, the relative sensitivity for CIN3^+^ lesions (with 95% confidence intervals), compared with a reference strategy, and the number of colposcopies needed to detect one lesion (CIN3^+^) were computed. The reference strategy at baseline (Strategy 1) refers to colposcopy for all HPV^+^ women with a positive triage cytology (any grade of cytological positivity) or with HPV16. The reference strategy at the 1‐year repeat (Strategy 5) refers to colposcopy for all women testing HPV^+^.

Differences in outcomes distribution among HPV types were tested using the Chi‐square test (*χ* ^2^) and the Fisher’s Exact test.

A *P*‐value <0.05 was considered statistically significant. SAS, v.9.4 (SAS Institute, Cary, NC, USA) was used for all analyses.

Core outcome sets and patient involvement are not relevant to this study.

## Results

This study included 145 437 women screened with an HPV test between January 2015 and December 2017 (Table S1). Overall, more than 90% of women were older than 35 years, but this proportion was lower for the Veneto cohort than for the other cohorts (86% versus 100%), as a pilot project involving women aged 25–64 years was conducted in Veneto starting in 2009,^27^ whereas the pilot phase in Emilia Romagna and Umbria only included women older than 35 years.^28^, ^29^

The results are presented divided by the specific time‐points of the screening episodes: baseline; 1‐year repeat and cumulative; follow up.

### Baseline

At baseline 9601 (6.6%) women were HPV^+^: 1.28% for HPV16, 0.41% for HPV18, and 4.91% for non‐16/18HPV types (Table 1).

**Table 1:** Comparison of the main results at baseline, at the 1‐year repeat and cumulative, according to HPV type at baseline

Screened women: 145 437					
HPV type	16	18	non16/18	All types	*P*‐value
***Baseline***					
Type of HPV (*n*)	1865	594	7142	9601	
Prevalence rates per 100 screened women	1.28	0.41	4.91	6.60	
Triage cytology					
Positive (ASC‐US^+^) cytology per 100 HPV^+^ (*n*)	44.1 (823)	34.3 (204)	32.8 (2342)	35.1 (3369)	<0.0001
Low‐grade cytology, % (*n*)	29.0 (540)	26.9 (160)	27.9 (1996)	28.1 (2696)	0.73
High‐grade cytology, % (*n*)	15.2 (283)	7.4 (44)	4.8 (346)	7.0 (673)	<0.0001
Histology					
Total histologies	757	189	2164	3110	
CIN3^+^ per 100 HPV^+^ (*n*)	7.9 (148)	2.0 (12)	1.3 (96)	2.7 (256)	<0.0001
CIN2^+^ per 100 HPV^+^ (*n*)	14.4 (269)	6.4 (38)	3.7 (263)	5.9 (570)	<0.0001
PPV for CIN3^+^ of biopsy per 100 histologies (*n*)	19.6 (148)	6.3 (12)	4.4 (96)	8.2 (256)	<0.0001
PPV for CIN2^+^ of biopsy per 100 histologies (*n*)	35.5 (269)	20.1 (38)	12.2 (263)	18.3 (570)	<0.0001
***1–year repeat***					
HPV tests	811	311	3821	4943	
HPV type x 100 HPV tests (*n*)	67.0 (543)	62.7 (195)	57.2 (2184)	59.1 (2922)	<0.0001
Cytology					
Total cytologies	526	187	2130	2843	
Positive (ASC‐US^+^) cytology per 100 cytologies (*n*)	27.8 (146)	28.3 (53)	25.3 (538)	25.9 (737)	0.28
Low‐grade cytology, % (*n*)	16.9 (89)	21.4 (40)	20.6 (438)	19.9 (567)	0.27
High‐grade cytology, % (*n*)	10.8 (57)	7.0 (13)	4.7 (100)	6.0 (170)	<0.0001
Histology					
Total histologies	527	188	2084	2799	
CIN3^+^ per 100 HPV tests (*n*)	4.2 (34)	2.6 (8)	0.8 (31)	1.5 (73)	<0.0001
CIN2^+^ per 100 HPV tests (*n*)	7.9 (64)	4.8 (15)	2.9 (112)	3.9 (191)	<0.0001
PPV for CIN3^+^ of biopsy per 100 histologies (*n*)	6.4 (34)	4.2 (8)	1.5 (31)	2.6 (73)	<0.0001
PPV for CIN2^+^ of biopsy per 100 histologies (*n*)	12.1 (64)	8.0 (15)	5.4 (112)	6.8 (191)	<0.0001
***Cumulative index***					
HPV^+^ at baseline	1865	594	7142	9601	
Histology					
Total histologies	1284	377	4248	5909	
CIN3^+^ per 100 HPV^+^ at baseline (*n*)	9.8 (182)	3.4 (20)	1.8 (127)	3.4 (329)	<0.0001
CIN2^+^ per 100 HPV^+^ at baseline (*n*)	17.9 (333)	8.9 (53)	5.3 (375)	7.9 (761)	<0.0001
PPV for CIN3^+^ of biopsy per 100 histologies (*n*)	14.2 (182)	5.3 (20)	3.0 (127)	5.6 (329)	<0.0001
PPV for CIN2^+^ of biopsy per 100 histologies (*n*)	25.9 (333)	14.1 (53)	8.8 (375)	12.9 (761)	<0.0001

CIN3^+^, CIN2^+^, cervical intraepithelial neoplasia grade 3+, 2+; HPV, human papillomavirus; ASC‐US, atypical squamous cells of undetermined significance; PPV, positive predictive value.

Overall, 35.1% of HPV^+^ women at baseline were positive at cytology triage: 44.1% for HPV16, 34.3% for HPV18 and 32.8% for non‐16/18HPV (*P* < 0.0001). These results were consistent among the three study regions.

An HG cytology was diagnosed in 15.2% (283/1865) of HPV16+ women versus 7.4% (44/594) of women with HPV18 and 4.8% (346/7142) of women with non‐16/18HPV (*P* < 0.0001).

At baseline, 3369 HPV^+^ cyto^+^ women were referred to colposcopy, and 3110 attended (attendance rate 92.3%). The detection rate of CIN3^+^ was 2.7% (256/9601): 7.9% among women with HPV16 infection (148/1865), 2.0% among women with HPV18 infection (12/594) and 1.3% among women with non‐16/18HPV infection (96/7142; *P* < 0.0001) (Table 1).

The detection of CIN3^+^ was mainly associated with an HG cytology (31.8%) versus 1.5% with LG cytology (*P* < 0.0001).

### One‐year repeat

Attendance with the repetition of HPV testing at 1 year was 79.3% (4943/6232). HPV positivity was recorded for 59.1% (2922/4943), with statistically significant differences according to HPV type at baseline: 67.0% for HPV16, 62.7% for HPV18 and 57.2% for non‐16/18HPV (*P* < 0.0001) (Table 1).

Among the women with positive HPV test at the 1‐year repeat, type‐specific persistence was recorded in 86.3% for HPV16 and 76.8% for HPV18, whereas repeat positivity for non‐16/18HPV types was observed in 95% of the cases.

The result of cytology at the 1‐year repeat is available for 2843/2922 (97%) women; 25.9% (737/2843) tested positive, without any statistically significant difference by HPV type at baseline. However, women with HPV16 at baseline and persistent HPV infection were twice as likely to have HG cytology compared with women with HPV18 or other HPV types (10.8% [57/526] compared with 7% [13/187] and 4.7% [100/2130], respectively; *P* < 0.0001).

Women with a positive HPV test at 12 months were referred to colposcopy; the attendance rate was 95.8% (2799/2922). The CIN3^+^ detection was 1.5% (73/4943), higher with positivity at baseline for HPV16 than other HPV types, i.e. 4.2% (34/811) versus 2.6% (8/311) for HPV18 infection and 0.8% (31/3821) for other HPV infection (*P* < 0.0001).

The detection of CIN3^+^ was 4.6% in women with persistent HPV16 infection at 1 year, whereas it was 1.4% in women with persistence of infection by HPV18 or other HPV types (*P* < 0.001).

### Cumulative

The cumulative (baseline plus 1‐year repeat) cytology positivity was 42.8% (4106/9601) and HG cytology was significantly higher with HPV16 infection at baseline (18.2%; 340/1865) than with HPV18 or other HPV types (9.6%; 57/594 and 6.2%; 446/7142, respectively) (*P *< 0.0001).

The cumulative detection of CIN3^+^ was 3.4% overall (329/9601) and it was two or three times greater for women with HPV16 infection than for women with HPV18 or non16/18HPV (9.8% versus 3.4% and 1.8%, respectively; *P* < 0.0001) (Table 1).

When women with multi‐infections were excluded, the baseline detection of CIN3^+^ was 8.3% in women with HPV16 alone, 1.5% in women with HPV18 alone and 1.3% in women with non‐16/18HPV. The cumulative detection of CIN3^+^ for the aforementioned groups was, respectively, 10.5%, 2.3% and 1.8%, similar to those observed for the three groups in the main analysis.

### Follow up

Follow‐up data were available for 2452/5148 (47.6%) women; most missing data refer to women whose follow up was not completed, mainly women in follow up after 1‐year HPV persistence. The HPV persistence was 57.5%; higher for HPV16 than for HPV18 or non16/18HPV (65.4% versus 57.1% and 55.4%, respectively; *P* < 0.0001).

The detection of CIN3^+^ at follow up was 0.8%, higher with HPV16 at baseline (2.5%) than with HPV18 or with non16/18HPV types (0.0% and 0.5%, respectively; *P* < 0.0001).

### Analysis by age

HPV positivity was 18.9% in the 25–29 age group and decreased at increasing age to 3.7% in the 55–64 group (Table 2). A different prevalence of the HPV types in relation to age was also observed; in particular, HPV16 infection was more frequently detected in the 25‐ to 29‐year‐old group than in the other groups: 4.6% versus 1.9% (30–44), 1.0% (45–54) and 0.6% (55–64), respectively (*P* < 0.0001).

**Table 2:** Comparison of HPV positivity at baseline and cumulative lesions (baseline + 1‐year HPV repeat), by HPV type and age class (data from Veneto and Umbria regions)

Age class			25–29 years		30–44 years		45–54 years		55–64 years		*P*‐value*
Screened women			*Total n* = 3441		*Total n* = 45 446		*Total n* = 41 388		*Total n* = 35 296		
			*n*	%	*n*	%	*n*	%	*n*	%	
Baseline	HPV^+^	Total	651	18.9	4002	8.8	2298	5.6	1294	3.7	<0.0001
		HPV 16	159	4.6	854	1.9	398	1.0	220	0.6	<0.0001
		HPV 18	35	1.0	253	0.6	164	0.4	72	0.2	<0.0001
		non16/18 HPV	457	13.3	2895	6.4	1736	4.2	1002	2.8	<0.0001
Cumulative			***n***	**‰**	***n***	**‰**	***n***	**‰**	***n***	**‰**	
	CIN3^+^ (per 1000)	Total	12	3.5	183	4.0	67	1.6	25	0.7	<0.0001
		HPV 16	8	2.3	109	2.4	32	0.8	12	0.3	<0.0001
		HPV 18	0	0.0	6	0.1	7	0.2	2	0.1	0.48
		non16/18 HPV	4	1.2	68	1.5	28	0.7	11	0.3	<0.0001
		*P*‐value**		0.002		<0.0001		<0.0001		<0.0001	
	CIN2^+^ (per 1000)	Total	37	10.8	427	9.4	159	3.8	51	1.4	<0.0001
		HPV 16	20	5.8	208	4.6	54	1.3	20	0.6	<0.0001
		HPV 18	1	0.3	24	0.5	14	0.3	5	0.1	0.04
		non16/18 HPV	16	4.6	195	4.3	91	2.2	26	0.7	<0.0001
		*P*‐value**		<0.0001		<0.0001		<0.0001		<0.0001	

CIN3^+^, CIN2^+^, cervical intraepithelial neoplasia grade 3+, 2+; HPV, human papillomavirus.

* Comparison by age class.

** Comparison by HPV type within each age class.

The cumulative detection of CIN3^+^ was higher in the 25–29 (3.5 per 1000 screened) and in the 30–44 age (4.0) groups and decreased to 1.6 in the 45–54 and 0.7 in the 55–64 age groups (*P* < 0.0001) (Table 2).

In the two younger age groups (i.e. 25–44 years), about two‐thirds of CIN3^+^ cases were associated with HPV16 (67% in the 2‐ to 29‐year‐olds and 60% in the 35‐ to 44‐year‐olds), whereas about half of the cases were associated with HPV16 (48% in both age groups) in older women, and non16/18 HPV types accounted for 42% and 44% of cases, respectively, in women 45–54 and 55–64 years old.

### Comparison of triage strategies

**Figure 2:**
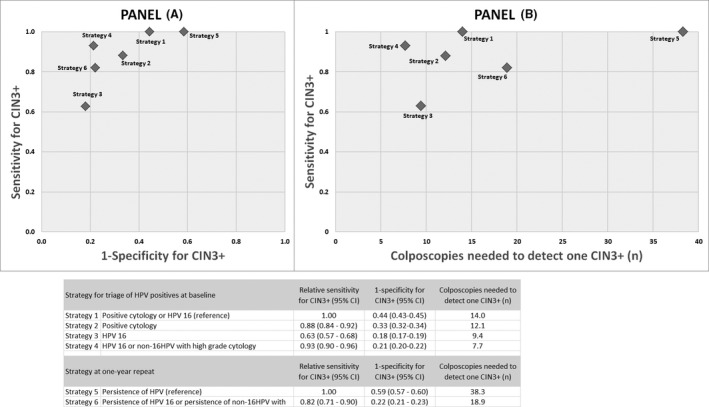
Comparison of triage strategies of positive HPV at baseline and 1‐year repeat: combination of sensitivity and specificity for CIN3^+^ (A) and sensitivity for CIN3^+^ and number of colposcopies needed to detect one lesion (B).

Figure 2 shows the combination of sensitivity and specificity for CIN3^+^ (panel A), and sensitivity and number of colposcopies needed (NNC) to detect one lesion (panel B) for various strategies at the baseline and at 1‐year repeat. Compared with the reference strategy (highest sensitivity) for the baseline (referral to colposcopy of women with positive cytology or HPV16), the strategy based on HPV16 alone (Strategy 3) would detect about two‐thirds of CIN3^+^ (relative sensitivity 0.63, 95% CI 0.57–0.68). On average, 9.4 colposcopies would be needed to detect one CIN3^+^, compared with 12.1 under the current strategy (positive triage cytology, Strategy 2). Referring to colposcopy, both HPV16 and non‐16HPV with high‐grade triage cytology (Strategy 4) would yield a sensitivity of 0.93 (95% CI 0.90–0.96), with the lowest NNC to detect one CIN3^+^ (7.7).

At the 1‐year repeat, referring to colposcopy women with a persistent HPV16 or a persistent non‐16HPV with positive cytology (Strategy 6) would miss 18% CIN3^+^ (relative sensitivity 0.82, 95% CI 0.71–0.90), compared with the (current) referral of all women with persistence of HPV (Strategy 5). However, the NNC to detect one CIN3^+^ would be 18.9 versus 38.3.

## Discussion

### Main findings

In this study we evaluated partial HPV16/18 genotyping as a triage biomarker for high‐risk HPV‐positive women identified within organised HPV cervical screening, in order to devise new strategies to improve its efficacy. This is especially important for the management of HPV‐positive women with negative cytology at baseline, which is particularly challenging.^30^

Infection by HPV16 was associated with greater detection of high‐grade cytology, viral persistence and CIN3^+^ development than any other HPV type (including HPV18) at all time‐points considered (baseline, 1‐year repeat, follow up). With all analyses, it emerged that HPV18 infection behaves more similarly to other high‐risk HPV types than to HPV16, in accordance with previously published results.^41^ This implies that the sensitivity and PPV values of HPV16/18 are considerably lower than those of HPV16 alone. Indeed, the HPV18 infection represents a peculiar situation due to its rather low risk for pre‐neoplastic lesions coupled with a high risk of cancer, especially the glandular type, which is more difficult to diagnose by cytological screening.

In particular, triage cytology at baseline was positive in almost half of women with HPV16, but in only one‐third of women with HPV18 or other HPV types. Further, about one in three of positive cytologies in HPV16 cases were HG, as compared with one in five with HPV18 and one in seven with non16/18HPV.

According to the Italian screening protocol, women who test HPV‐positive/cytology‐negative are referred to repeat HPV testing after 1 year, followed by colposcopy in the case of positivity (irrespective of cytology result). Overall, nearly 60% of these women are still HPV‐positive, and viral persistence was greater in women with HPV16 than with other HPV type infection at baseline (up to 10 percentage points than for HPV18), without significant differences by age. Longer intervals between baseline and HPV testing repetition, as well as risk stratification by molecular biomarkers, have been proposed.^14^, ^16^

The role of HPV16 also emerged when considering histologically confirmed high‐grade lesions. At baseline, the overall detection of CIN3^+^ was 2.7%, with a PPV for HPV^+^/cyto^+^ at colposcopy of 8.2%. The detection of CIN3^+^ among women infected by HPV16 was 7.9%, with a PPV of 19.6%, whereas HPV18 infection had a lower association with CIN3^+^ (CIN3^+^ detection 2.0%, PPV 6.3%), closer to non16/18HPV types (CIN3^+^ detection 1.3%, PPV 4.4%).

At the 1‐year recall, the detection of CIN3^+^ decreased, as expected, but the association with HPV16 was confirmed (PPV of 6.4% versus 4.2% for HPV18 and 1.5% for other HPV types).

As expected, the detection of CIN3^+^ further decreased at follow up, but also in this case, the relative weight of HPV16 compared with the other HPV types was further strengthened.

These findings highlight that partial HPV16 genotyping may play a role in improving immediate and short‐term triage, whereas HPV18 seems to behave much more similarly to the other HPV types and does not provide additional stratification. Nonetheless, as several studies indicate that HPV18 persistence is associated with an increased long‐term risk,^31^, ^32^, ^33^, ^34^, ^35^, ^36^, ^37^, ^38^, ^39^, ^40^, ^41^ it is very important to follow up these women appropriately.

### Strengths and limitations

Our findings derive from routine clinical practice within quality‐assured population‐based organised cervical cancer programmes. Attendance was 92.3% at immediate colposcopy and 95.8% at colposcopy after 1‐year HPV persistence, and compliance with HPV repetition at 1 year was 79%. Moreover, the main results showed high levels of consistency among the three study regions, supporting generalisability to Italy as a whole, and possibly to other European countries.

A limitation of our study is that not all the analyses performed for the baseline and 1‐year recall could be performed for the follow‐up outcomes (especially for cytology) due to partial availability of the data. Moreover, as not all the women underwent colposcopy, we cannot exclude some risk of verification bias.

### Interpretation

The close association between HPV16 infection at baseline and CIN3^+^ at all three time‐points could be exploited within triage protocols based on partial HPV16 genotyping, to provide a more efficient risk stratification and to reduce the number of colposcopies needed to detect a lesion, as suggested by the recent literature.^42^

Based on these findings, Figure 2 provides a separate analysis of the role of the strategies for CIN3^+^ at baseline and the 1‐year repeat.

- At baseline, the most sensitive strategy (1, positive cytology or HPV16) does not seem to offer the best balance between lesion detection and the need for colposcopies. On the other hand, Strategy 4 (HPV16 or non16‐HPV with HG cytology), which combines reasonable sensitivity with a considerable decrease in the required number of colposcopies, appears to be the most attractive.
- At 1‐year recall, Strategy 6 (HPV16 persistence or non16‐HPV with HG cytology), compared with the reference, which is the current standard in Italy, shows a saving of about 50% in the number of colposcopies needed and a reasonable loss of sensitivity.

Previous studies have shown other strategies to be effective. In particular, data from the ATHENA study suggested referring to colposcopy women who were ASC‐US/HPV‐positive and LSIL or more severe cytology, irrespective of HPV result, as well as all women who were cytology‐negative/HPV16/HPV18‐positive.^43^ This strategy is very similar to the one presented in our analysis for baseline, but a direct comparison is not possible, as we have analysed HPV16 separately from the other HPVs. As the birth cohorts involved in the HPV vaccination campaigns will come up for cervical screening, the clinical relevance of partial HPV16 genotyping will decrease, and different triage strategies will be necessary. We^44^ and others^41^ have reported that other HPV types are associated with increased risk for CIN3^+^ development, in particular HPV33, HPV 35, HPV31; additional typing might therefore be useful, especially in vaccinated cohorts.

Given the important role of HPV16 in detecting CIN3^+^ lesions, as highlighted in our study, it appears that a strategy in which HPV16 positivity is utilised as a stratification tool may answer a currently posed question as to whether it is necessary to detect all CIN3^+^ or just those that are most likely to progress.

## Conclusions

The results of this study demonstrate that within organised cervical screening the combination of partial genotyping and HG cytology has a potential for better stratification after HPV positivity.

### Acknowledgements

The authors wish to thank Dr. Luca Weis for assistance in the analysis of data and helpful discussion.

### Disclosure of interests

The authors declare no potential conflicts of interest. Completed disclosure of interests forms are available to view online as supporting information.

### Contribution to authorship

SG conceived the study, collected and analysed the data and drafted the manuscript. MZ, CC, RF, BP locally coordinated the screening activities and retrieved the data. JB and MZ performed the statistical analyses and reviewed the manuscript. SG, DG, HF, GS, SB, CF, EC performed HPV and/or cytological analyses. ADM supervised the planning design and the data analyses and reviewed the manuscript. All authors read and approved the final version of the manuscript.

### Details of ethical approval

The study was performed in the context of a clinical service review and therefore ethical approval was not required.

### Funding

None.

## Supporting information


**Table S1.** Regions involved in the study, with study periods, number of screened women overall and by age, and proportion of positive HPV tests.

Supplementary material

Supplementary material

Supplementary material

Supplementary material

Supplementary material

Supplementary material

Supplementary material

Supplementary material

Supplementary material

Supplementary material

Supplementary material

Supplementary material

Supplementary material
