# Supplementary material for: Clinical relevance of partial HPV16/18 genotyping in stratifying HPV‐positive women attending routine cervical cancer screening: a population‐based cohort study
Source: BJOG. 2021 Jan 12;128(8):1353–62. doi: 10.1111/1471-0528.16631 (PMC8248328; doi:10.1111/1471-0528.16631)
Supplement: Supplementary file 1 — Table S1. Regions involved in the study, with study periods, number of screened women overall and by age, and proportion of positive HPV tests. [file BJO-128-1353-s007.pdf]

Table S1. Regions involved in the study: study periods, number of screened women overall and by age, and proportion of positive HPV tests

Area	Period	All women	25-34 years	35+ years	HPV+
Veneto	May 2015 – May 2017	88,509	12,436 (14.1%)	76,073 (85.9%)	5,919 (6.7%)
Umbria	January 2015 – December 2016	37,082	39 (0.1%)	37,043 (99.9%)	2,326 (6.3%)
Emilia-Romagna	September 2016 – December 2017	19,846	5 (0%)	19,841 (100%)	1,356 (6.8%)
Total		145,437	12,480 (8.6%)	132,957 (91.4%)	9,601 (6.6%)

Abbreviation: HPV = human papillomavirus
